# Large Language Models for Mental Health Prediction: Scoping Review of Bias and Clinical Utility Documentation in 2019-2024

**DOI:** 10.2196/88082

**Published:** 2026-08-13

**Authors:** Clémentine Bleuze, Karën Fort, Vincent P Martin, Aurélie Névéol

**Affiliations:** 1 Loria, Université de Lorraine, CNRS, Inria, Loria Campus Scientifique, BP 239, Nancy, F-54000, France, 33 3-83-59-20-20; 2 LISN, Université Paris-Saclay, CNRS, Orsay, France

**Keywords:** natural language processing, large language models, mental health, bias, clinical utility

## Abstract

**Background:**

A growing body of literature leverages large language models (LLMs) to make mental health predictions. However, these models are prone to bias, and studies to validate their clinical utility are lacking.

**Objective:**

This scoping review aims to uncover bias and clinical utility limitations stemming from the methodological design of LLM-based mental health predictive systems. In addition, it intends to document the level of self-reflection about bias and clinical challenges reported by authors in their own work.

**Methods:**

This work follows the PRISMA-ScR (Preferred Reporting Items for Systematic Reviews and Meta-Analyses extension for Scoping Reviews) guidelines and was registered online. Eligible studies were original research articles in English published between 2019 and 2024, using LLMs to detect mental health conditions in nonsynthetic textual data. The search was conducted in 5 scientific databases (PubMed, Web of Science, IEEE Xplore, ACM Digital Library, and ACL Anthology) with queries associating keywords related to “Mental Health,” “Large Language Models,” and “Prediction.” We extracted both methodological information about the included studies and authors’ statements relevant to issues of bias and clinical utility. This extraction was based on a framework screening the entire pipeline of development of LLMs with applications in mental health: research design and selection, data collection, outcome definition, model development, and postdeployment considerations. Statistical description of the retrieved entities, as well as thematic coding, was performed for analysis.

**Results:**

A total of 2472 articles were identified, of which 263 (10.6%) were assessed for eligibility, and 201 (8.1%) were included in the review. Included studies were mostly recent, indicating a growing interest in the use of LLMs for mental health predictions. Our analysis revealed that a majority of studies share similar methodological choices along their development pipeline: most of them focus on depressive disorders identified via processing user texts on social media, mainly with the use of nonspecialist LLMs derived from BERT (Bidirectional Encoder Representations from Transformers). Following previous works on these matters, we highlighted how these choices may hinder the clinical relevance and fairness of the envisioned systems. Similarly, we found that 164 (81.6%) studies mention themes related to bias and clinical utility; however, most of the discussion revolves around data-centered issues. Only 41 (20.4%) articles mention themes associated with at least 3 out of 5 pipeline steps, suggesting a limited appropriation of the notions of bias and clinical utility in such a sensitive context as mental health analysis.

**Conclusions:**

Bias and clinical utility are lightly covered in the field of LLM-based mental health prediction research as of 2019‐2024. In-depth approaches involving interdisciplinary teams of clinicians and natural language processing specialists are needed to ensure technical soundness, clinical relevance, and fair outcomes for potential users.

## Introduction

### Background

Biomedical natural language processing (NLP) methods aim to support clinical research and practice, with suggested uses spanning information retrieval, medical documentation generation, and patient data analysis [1,2]. As language has been studied as a marker for conditions such as depression [3], schizophrenia [4,5], and Alzheimer disease [6], mental health–oriented tasks have become popular in NLP. Notably, methods to analyze patient-authored texts or medical records as indicative of a given condition or symptom have been developed, with the underlying hypothesis that this could translate into clinically useful tools for diagnosis or large-scale screening [7-9]. A number of these methods are now based on large language models (LLMs), which recently garnered global attention from the public, industrials, and researchers for their assumed general language capabilities.

However, it is increasingly documented that LLMs can produce unreliable results and potentially harm users by perpetuating and amplifying bias [10-14], which has been defined as “the presence of systematic errors or disparities within decision-making processes that disproportionately affect specific subgroups” [11]. While current medical practice is admittedly not exempt from bias itself [15], automated systems such as LLMs amplify existing stereotypes, which can lead to unjustified differences in simulated diagnoses and treatment plans for vignettes of patients with different ethnicities and genders [13]. Relatedly, it has been reported that male profiles are overgenerated by LLMs (regardless of the true prevalence of the considered condition) both in English and in French [13,14]. Despite substantial research efforts, no reliable mitigation technique has been proved to fully resolve these issues [16,17], which may be intrinsic to LLMs [18].

Moreover, existing evaluation settings for biomedical LLMs are limited [10,19] and real-world impact studies are still lacking in the NLP community [20], leaving many ethical and regulatory challenges unaddressed [21]. As a result, it is still unclear to what extent these LLM-based systems are clinically “[useful] in improving patient outcomes, informing clinical decision-making, and optimizing health care resources” [22] in mental health. In addition, computer scientists and NLP engineers creating such systems may lack the medical expertise needed to make accurate design choices. It seems therefore crucial to methodically audit LLM-based systems both for bias and clinical utility before considering downstream clinical deployments.

Existing reviews have investigated the applications, benefits, and risks of LLMs in health care [23] and mental health [24-26], highlighting general ethical challenges such as data privacy, fairness and bias, clinical integration, and ethical governance. There is, however, a lack of literature that explicitly connects these different issues and systematically assesses them throughout the development steps of the considered systems, taking a distance from indicators of (possibly ill-defined [27]) performance. Notably, although they are crucial to understand downstream risks, existing bias studies in health-related scenarios generally posit bias as a single-dimension construct (eg, gender or racial bias) [17,28], using LLMs as readily available tools whose development is left unquestioned. Yet the decision-making processes from which bias can stem are numerous, which calls for a bias investigation throughout the entire development pipeline of the considered system [29,30]. Similarly, the limitations and barriers to the clinical implementation of NLP technologies [19,31] tend to be listed as post hoc challenges of the systems rather than connected with underlying design choices. In this review, we consider bias and clinical utility not only to be both related and important issues, but to be complementary when it comes to implementing (or envisioning the possible implementation of) any automated system in real-world health care scenarios. Indeed, both these dimensions (How biased will the final system be? How clinically useful?) depend greatly on methodological choices spread throughout the development of the system.

Building on these considerations, we propose to study bias and clinical utility as 2 intricate dimensions rooted in methodological choices, with expected downstream social impact on users. To our knowledge, this is the first such extensive, large-scale joint analysis of bias and clinical utility for predictive mental health applications based on LLMs.

### Objectives

The objectives of this work are 2-fold. First, we wish to broadly describe the methodological decisions adopted in publications on LLM-based mental health prediction. This will lead us to analyze possible bias and barriers to clinical utility stemming from these methodological choices, at each step of the system’s development pipeline.

Second, we intend to document the awareness of researchers on matters of bias and clinical utility, as indicated by relevant reported considerations in these same publications. Indeed, increased global research attention on these issues does not necessarily translate into actionable plans for other researchers [32]. Furthermore, propensity to bias is still absent from reference evaluation benchmarks used to evaluate these systems, which shows limited appropriation of these issues within the community.

Drawing on these insights, we mean to foster discussions as to the extent to which it is currently possible, safe, and desirable to integrate LLMs into clinical routine and mental health care in particular.

## Methods

### Registration and Protocol

This scoping review was preregistered in the Open Science Framework registry [33]. We followed the PRISMA-ScR (Preferred Reporting Items for Systematic Reviews and Meta-Analyses extension for Scoping Reviews) guidelines [34] (refer to Checklist 1).

### Eligibility Criteria

We analyzed original research articles published in conferences or journals between January 2019 (to account for the release of BERT [35]) and December 2024. Eligible studies had to (1) assess the presence or severity of a mental health condition; (2) use, for that purpose, an NLP system comprising at least one LLM; and (3) use nonsynthetic textual samples as input data, possibly along with synthetic samples or other modalities. We excluded preprints, reviews, and studies which were not openly available online, as well as papers not written in English.

Importantly, following criterion (1), articles claiming to perform only stress detection or sentiment analysis were not considered eligible. Regarding criterion (2), as it can be underspecified in the literature which models fall under the designation of “LLMs,” we followed the recommendation of Rogers and Luccioni [36] to provide an explicit working definition. In accordance with standard considerations [37], we therefore called LLMs NLP tools that (1) model and can generate text, (2) receive large-scale pretraining, (3) can be used for transfer learning, and (4) rely on the Transformer architecture [38]. Notably, this definition includes both encoder models such as BERT [35] and generative decoder models such as GPT [39], in line with existing reviews about applications of LLMs to mental health [24]. Finally, although criterion (3) allows for the inclusion of papers developing systems for audio or imaging data, our analysis was oriented toward the LLM-relevant part of these systems, that is, the one processing text. Besides, even if synthetic datasets are increasingly praised for clinical research [40], we wanted to focus in this review on systems that fulfill at least partially expectations of pending deployment—generally comprising testing in realistic conditions which are, up to now, only imperfectly proxied by generated corpora [41,42]. In addition, our framework comprises a step which focuses on the included data, considering demographics of included participants, which is not applicable to synthetic datasets.

### Search Strategy

In order to provide a wide coverage of relevant publications, we searched 5 databases spanning biomedical, NLP, and more general AI-related literature: MEDLINE (PubMed), Web of Science, IEEE Xplore, ACM Digital Library, and the ACL Anthology. All searches were performed on January 20, 2025, with queries following the template (terms related to “MENTAL HEALTH”) AND (terms related to “LARGE LANGUAGE MODELS”) AND (terms related to “PREDICTION”). The queries were crafted following multiple search rounds and designed to minimize false negatives; hence, the large number of allowed terms in each keyword group (request details are presented in Multimedia Appendix 1). When allowed by the database search engine, we used filters for date, publication language, and publication type according to the eligibility criteria listed above.

### Screening Process

The initial set of records identified via database search was loaded into the Rayyan software (Mourad Ouzzani) [43]. A sample of 100 records was first screened based on title and abstract by 3 reviewers (CB, AN, and KF) independently. Interannotator agreement was high, with pairwise Cohen κ [44] scores ranging from 0.654 to 0.807 [45], while resulting discussions helped clarify the screening strategy (see “Eligibility Criteria” section). Subsequently, the first author (CB) screened the remainder of records, as well as the full text of all the eligible studies before conducting extraction. Exclusion reasons and record counts at every step are detailed in the flowchart. Remaining doubts were addressed through discussion between authors.

### Data Extraction

In accordance with our research objectives, we extracted 2 types of information in the reviewed papers: methodological information (what do the authors do?), and authors’ self-reflections and acknowledgments linked with bias and clinical utility (what do the authors say about what they do?). The full-text PDFs of included studies were loaded into Zotero (Corporation for Digital Scholarship) [46] for reading, alongside an auxiliary spreadsheet document for qualitative entity extraction performed by CB.

To our knowledge, there is no existing framework specifically designed to study both bias and clinical utility in the conception pipelines for LLMs applied to mental health. However, 2 previous approaches, which we deem complementary to analyze bias and clinical utility at every step of the development pipeline of an LLM-based system for mental health prediction, were proposed.

On the one hand, Hovy and Prabhumoye [29] identified 5 sources of bias in NLP systems: the data, the annotations made on the data, the input representations fed to NLP models, NLP models themselves, and larger research design choices such as the treated language. On the other hand, Chen et al [30] sketch a 5-step pipeline of ethical stakes for machine learning (ML) in health care, considering (1) the unequal distribution of funding and research attention between health issues with regards to the share of the world population that they affect (problem selection), (2) nonrepresentative data (data collection), (3) imperfect medical proxies for target outcomes (outcome definition), (4) ML models optimization parameters potentially exacerbating bias (algorithmic development), and (5) blindness to issues such as generalizability to various clinical settings, downstream impact assessment or regulatory compliance before real-life system deployment (postdeployment considerations).

**Figure 1.**
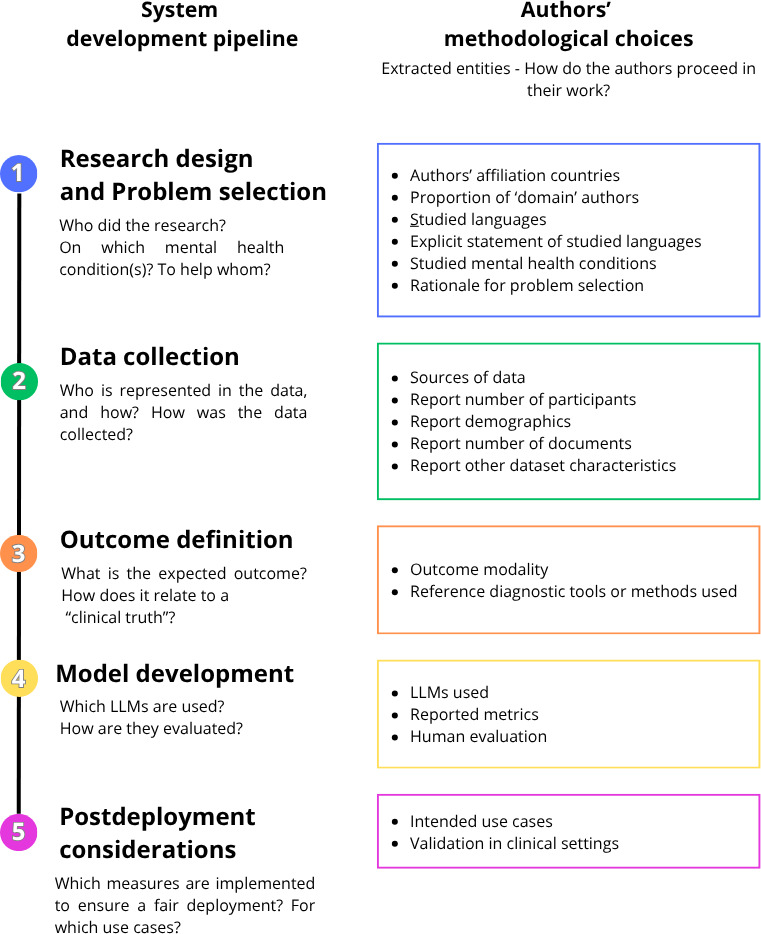
Left: our proposed 5-step pipeline for analyzing bias and clinical utility in large language model–based mental health prediction systems. Right: entities extracted in papers, in accordance with each pipeline step. LLM: large language model.

Inspired by these 2 previous approaches, we propose a 5-step framework (Figure 1) to study both bias and clinical utility within the selected articles. We identify key arguments of each work, and define qualitative entities to extract from papers for future analysis. For instance, Chen et al [30] discuss the importance of measuring performance metrics across demographic groups to ensure fair treatment among patients, which we operationalize as a binary entity “per-group performance” to be set to “true” when authors report disaggregated results (eg, against sex or gender, race or ethnicity, or other sensitive attributes). This entity relates indeed both to bias (as some groups may be discriminated against if the system performs poorly for them) and to clinical utility (as clinical outcomes are expected to be fair among patients). We redirect the reader to Multimedia Appendix 2 for more details on our analysis framework design and the operationalization of entities.

We also collected sentences from the included articles that refer explicitly to any sort of bias or to elements related to the clinical utility of the developed system, as a proxy for self-reflection of researchers regarding these matters. We extracted these statements independently from the arguments they defend (eg, recognizing the presence of bias in presented results vs stating that bias has been mitigated), within our extraction framework.

### Data Analysis

We performed the analysis of the extracted data in a Jupyter Notebook (Project Jupyter) environment, using Python libraries (Guido van Rossum). When applicable, usual descriptive statistics (distribution of values, mean value, range, and so on) were computed. In some cases when necessary conditions were met, we modeled the effect of categorical variables (eg, the effect of medical affiliation on the studied conditions) using chi-squared contingency tests (*χ*^2^) computed with the *SciPy* Python package (without correction). The entities for which full sentences or noisy outputs were extracted were further analyzed by clustering the extracted data into meaningful categories, in an iterative and inductive aggregation process inspired by grounded theory [47]. For authors’ self-reflections about bias and clinical utility, we used the same approach to map the highlighted sentences to themes. The final results (extracted entities or categories obtained after coding for each paper) following data analysis are presented as a spreadsheet in Multimedia Appendix 3.

## Results

### Study Selection

**Figure 2.**
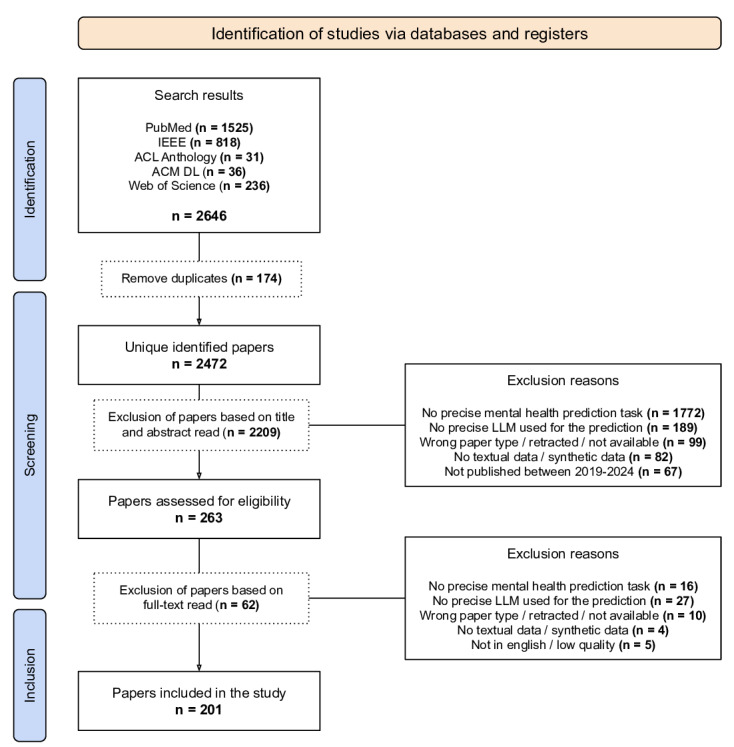
PRISMA (Preferred Reporting Items for Systematic Reviews and Meta-Analyses) flowchart.

We initially identified 2646 candidate records in databases (search performed on January 20, 2025). After deduplication, 2472 unique papers were screened for eligibility based on title and abstract. The remaining 263 papers were then screened based on full-text read. Finally, 201 (8.1%) studies were included in this review. The PRISMA (Preferred Reporting Items for Systematic Reviews and Meta-Analyses) diagram of the process is detailed in Figure 2, and the full list of included papers is available in Multimedia Appendix 4.

Included studies (N=201) are mostly recent: 149 (74.1%) are published in 2023‐2024, 46 (22.9%) in 2021‐2022, and only 6 (3%) in 2019‐2020. Most of the retrieved studies are sourced from IEEE Xplore (124/201, 61.7%) and PubMed (57/201, 28.4%). The ACL Anthology, the Web of Science, and the ACM Digital Library, respectively, raised 10 (5%), 7 (3.5%), and 3 (1.5%) papers.

### Global Trends in Bias and Clinical Utility Report by the Authors

We found mentions of bias and clinical utility-related themes in 164/201 (81.6%) papers. The pipeline step that triggered the most discussion is that of data collection (107/201, 53.2%), followed by model development (69/201, 34.3%), and problem selection and research design (60/201, 29.8%). Themes associated with outcome definition and postdeployment considerations were only evoked in, respectively, 45/201 (22.4%) and 31/201 (15.4%) papers. In 77/201 (38.3%) papers, the discussion was restricted to a single step of the pipeline. However, 46/201 (22.9%), 26/201 (12.9%), and 10/201 (5%) papers evoked themes associated with 2, 3, and 4 of these steps. A total of 5 (2.5%) papers mentioned considerations from all 5 steps of the pipeline [48-52].

### Bias and Clinical Utility Along the System Development Pipeline

#### Overview

In this section, we describe entities extracted from the included studies, as illustrated in Figure 1. For each of the 5 steps of pipeline design potentially subject to biases and clinical utility limitations, we both report the extracted entities and the relevant themes explicitly mentioned by authors about their work under a paragraph entitled “Mentions of Bias and Clinical Utility–Related Themes.”

#### Step 1: Research Design and Problem Selection

##### Overview

The step of research design and problem selection raises the following questions: who did the research? On which mental health conditions? To help whom?

##### Affiliation Countries of Authors

Forty-five distinct countries are represented in author affiliations, spanning all 6 continents. There is, however, a concentration of publications with author affiliations in China (43/201, 21.4%), the United States (42/201, 20.9%), and India (32/201, 15.9%). International collaborations are present in 54 papers (26.9%), with up to 4 different countries of affiliation in a single publication.

##### Domain Affiliation of Authors

A total of 76 (37.8%) papers comprise at least one author declaring an affiliation related to the medical domain (domain author). The average ratio of domain authors over all authors within a paper is 0.22, with a median value of 0.0 (IQR 0.33) and an SD of 0.34. A total of 20 (10%) papers have a domain-to-all ratio of 1.0, indicating that they are written only by domain-affiliated authors.

##### Languages

**Figure 3.**
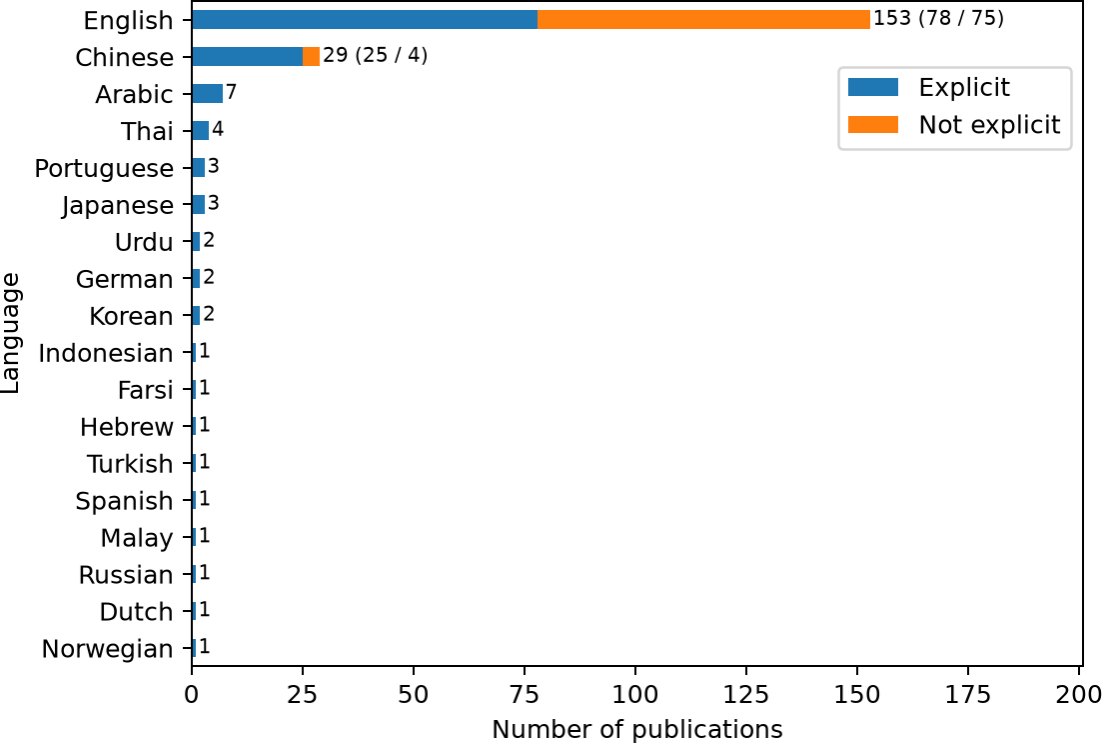
Distribution of the languages studied in the included papers (ie, the languages of the data the systems make predictions about). For each language, it is also indicated whether it is explicitly mentioned by the authors in the publications.

As for the languages the authors work with (ie, those of the processed data), 18 distinct languages are identified (Figure 3). While English is overtly predominant (153/201, 76.1%), other treated languages include Chinese (29/201, 14.4%), Arabic (7/201, 3.5%), Thai (4/201, 2%), Portuguese (3/201, 1.5%), Japanese (3/201, 1.5%), and others. Of the publications on English and Chinese, respectively, 75/153 (49%) and 4/29 (13.8%) omit to state the treated language explicitly; therefore, this information has to be deduced based on the models or resources used.

##### Studied Mental Health Conditions

Fifteen mental health disorder groups (mapped to condition groups found in the *DSM-5* [*Diagnostic and Statistical Manual of Mental Disorders, Fifth Edition*] [53], as well as a generic category of “Suicidal Risk” for papers studying suicidal ideation, behavior, or attempts) are studied in the selected articles (Table 1). A majority of the reviewed papers work on predicting depressive disorders (148/201, 73.6%), significantly more so when the papers comprise no domain author (*χ*^2^_1_=10.95, *P*<.001). Other prevalent selected conditions include suicidal risk (47/201, 22.9%) and anxiety disorders (17/201, 8.5%), with a variety of other condition groups marginally represented. Besides, while 175 (87.1%) papers focus on a single condition, 26 (12.9%) papers study at least 2 of them, with a maximum coverage of 10 conditions in a single paper [54].

**Table 1.** Distribution of mental health disorder groups among studies (some studies include multiple disorder groups).

Mental health disorder group	Paper count, n (%)
Depressive disorders	148 (73.6)
Suicidal risk	47 (23.4)
Anxiety disorders	17 (8.5)	
Trauma and stressor-related disorders	14 (7)	
Bipolar and related disorders	13 (6.5)	
Schizophrenia spectrum and other psychiatric disorders	11 (5.5)	
Attention-deficit hyperactivity disorder	10 (5)	
Personality disorders	8 (4)	
Feeding and eating disorders	7 (3.5)	
Major and mild neurocognitive disorders	5 (2.5)	
Autism spectrum disorders	5 (2.5)	
Intellectual disabilities	3 (1.5)	
Obsessive-compulsive and related disorders	2 (1)	
Dissociative disorders	1 (0.5)	
Other (perinatal psychiatry)	1 (0.5)	

##### Rationale for Problem Selection

Three major themes are identified which relate to the conditions under study or to mental health in general: negative impacts on individuals (165/201, 82.1%; eg, “Mental illness is [...] significantly impacting the lives of individuals across diverse communities” [54]), a high or increasing prevalence (170/201, 84.6%; eg, “Suicide is one of the main causes of death in the world” [55]), and shortcomings of the “traditional” medical system to efficiently tackle the problem (127/201, 63.2%; eg, “Constrained clinician time and a strong focus on anticancer treatment may contribute to the insufficient identification of patients at risk for depression” [56]). Besides, 9/201 (4.5%) papers explain that their study takes place in the context of shared tasks organized by the scientific community. These tasks are hosted by labs or workshops at conferences such as CLEF, CLPsych, ACL, and IEEE BigData.

##### Mentions of Bias and Clinical Utility–Related Themes

Overall, 60/201 (29.8%) papers mention themes related to the step of research design and problem selection, and 38/201 (18.9%) mention language and culture as a potential bias source related to the step of problem selection. This comprises both acknowledgments that results on English may not generalize to other languages, and cases where authors highlight that they work on an underserved language. Others (22/201, 10.9%) explicate how population disparities related to the condition under study oriented the design of their work toward more vulnerable groups. For instance, some researchers study mental health issues of pregnant or postpartum women [49], US service members and veterans [57], children or adolescents [58], patients with cancer [59], and so on. As for the choice of a condition, 4 (6.7%) papers claim to tackle a research gap, such as Juhng et al [52] who state that “much less work in the NLP community has focused on detecting anxiety disorders as has been done for depressive disorders.” Finally, 1 (0.5%) paper explicitly acknowledges the “absence of direct involvement with mental health experts,” which we map to the theme of workforce diversity.

### Step 2: Data Collection

#### Overview

The step of data collection raises the following questions: who is represented in the data, and how? How was the data collected?

#### Data Sources

We identify 3 major data provenances for the datasets used in the reviewed papers. First, posts from social media and online forums are used in 133/201 (66.2%) papers, under the frequent rationale that it consists of large-scale, spontaneous, and anonymous testimonies of users about their mental health. This data source is significantly more adopted in papers comprising no domain authors (*χ*^2^_1_=23.57, *P*<.001). The most prevalent sources within this category are Reddit (Reddit, Inc; 72/201, 35.8%), Twitter (X Corp; 44/201, 21.9%), and Sina Weibo (Weibo Corporation; 11/201, 5.5%). Data collections such as those provided in the CLEF conference for eRisk labs constitute a popular example of such social media datasets (see Textbox 1). Second, 62/201 (30.8%) papers make use of data collected from participants during clinical or semiclinical interactions. This comprises interview transcripts (eg, Patient Health Questionnaire (PHQ)–led interviews in the distressed analysis interview corpus and extended-distressed analysis interview corpus [60], refer to Textbox 1), narrative transcripts provided by participants about their medical experiences, texts entered on mental health monitoring apps, and therapy transcripts. Third, electronic health records of patients coming from different sites are used in 10/201 (4.9%) papers. Among them, one paper uses publicly available ScAN [61] (from MIMIC-III [62]), while the others rely on private datasets accessed through their institutional affiliations. A total of 2 (1%) papers gather multisite records, while the others are from a single source.

Textbox 1. **Erisk and the Distressed Analysis Interview Corpus (DAIC): 2 common datasets for automated mental health prediction.**

Erisk labs have been held at CLEF since 2017. Each edition comprises one or more tasks designed to “explore issues of evaluation methodology, effectiveness metrics, and other processes related to early risk detection” [63]. As of 2025, while multiple tasks have been proposed (anorexia and related eating disorders, self-harm, pathological gambling), the major focus has been on depression detection. Notably:

- A first dataset for the early detection of depression was used and extended throughout 2017 (task 1), 2018 (task 1), and 2022 (task 2) editions [63-65]. It initially consisted of 531,000 Reddit (Reddit, Inc) posts [66] collected from 892 Reddit users (137 depressed and 755 control) following a template-based heuristic and manual check. “Depression” posts are those of users who unambiguously stated their diagnosis (eg, “I was diagnosed with depression,” but not “I think I have depression”), while “Control” posts were those of random users as well as some posting in the r/Depression forum without explicit diagnosis statements.
- A second dataset was constituted for measuring the severity of the signs of depression in 2019 (task 3) and extended for 2020 (task 2) and 2021 (task 3) [67-69]. It contains the entire Reddit posting history of 20 users (up to 90 in 2021) who agreed to fill BDI-based questionnaires as ground-truth data. Lab participants thus have to predict the users’ scores for each item, as well as an overall depression assessment score, eventually mapped to 1 of 4 coarse-grained categories (minimal, mild, moderate, and severe depression).

The DAIC is a collection of 621 clinical interviews designed to “support the diagnosis of psychological distress conditions such as anxiety, depression, and post traumatic stress disorder” [60]. It comprises 4 subcorpora corresponding to distinct interview setups: face-to-face, teleconference, Wizard-of-Oz (WOZ, ie, interviews are led by a human-controlled virtual interviewer), and automated (ie, interviews are led by a fully automated virtual agent). The corpus data consist of audio and video recordings of interviews, partial transcriptions, and both verbal and nonverbal annotations. Interviewees consisted of veterans of the US armed forces and voluntary participants recruited via online ads in California, 397/621 (63.9%) of whom had their interview tagged as “distressed.” An extended version (E-DAIC) has since been constituted for the AVEC workshop at ACM MM 2019, although documentation is still lacking [70].

#### Reporting of Participants Count and Demographic Characteristics

We find that 122/201 (60.7%) papers omit to provide the number of participants included in the datasets they use in their work. When that count is disclosed, we find both very small (eg, 22 participants in the study by Li et al [71]) and very large datasets (eg, more than 43,000 patients in the study by Meng et al [72]). Demographic information about participants is reported in 43/201 (21.4%) papers. More precisely, information can be found about their age (35/201, 17.4%), their sex or gender (32/201, 15.9%), their race or ethnicity or cultural background (11/201, 5.5%), their education level or academic status (9/201, 4.5%), and other attributes such as marital status, household income, or additional medical information (9/201, 4.5%).

#### Reporting of Documents Count and Other Dataset Characteristics

Document counts (ie, the data samples which are fed to the model, such as social media posts or clinical notes) are reported in 172/201 (85.6%) papers. We note that this document count ranges from dozens of documents (eg, the study by Hayati et al [73]) to several hundred thousand documents (eg, the study by Feng et al [74]). Details about the time of data collection, class size (eg, the number of data samples tagged with or without depression), or outcome-related statistics (eg, the average anxiety score in a questionnaire-based dataset) are reported by the authors in 127/201 (63.2%) papers.

#### Mentions of Bias and Clinical Utility–Related Themes

We find mentions related to the step of data collection in 107/201 (53.2%) papers, making it the most discussed pipeline step with regard to bias and clinical utility. A total of 60/201 (29.8%) studies discuss the challenge of class imbalance (ie, when target labels are unevenly distributed in the data), which can lead to biased results. This imbalance is typically mitigated by authors using sampling or weighting techniques to avoid negative impacts on model performance. Representation bias mentions (ie, when the attributes of the people represented in a dataset mismatch those of the target population) are found in 52/201 (25.9%) papers and linked with diverse methodological (eg, site of collection and sampling method) and sociodemographic features (eg, gender, race and ethnicity, and social status). Other themes relate to the challenges of dealing with a small dataset (22/201, 10.9%), preexisting bias (3/201, 1.5%) in the data (eg, Hutto et al [51] acknowledge as a limitation that “bias may be introduced by the author of the [medical] note” and static datasets (3/201, 2.8%) which can only account for a person’s condition at a given point in time, whereas clinical decisions are usually made based on a person’s medical history.

### Step 3: Outcome Definition

#### Overview

The step of outcome definition raises the following questions: what is the expected outcome? How does it translate to clinically actionable information?

#### Outcome Modalities

Different outcome modalities are found in the reviewed studies, with sometimes several ones in the same paper. In 146/201 (72.6%) papers, simple binary labels are assigned to data samples for the condition under study (eg, “depression” vs “no depression”). In 46/201 (22.8%) papers, severity degrees are instead estimated on ordinal, qualitative scales typically comprised 3 to 4 values (eg, “no depression,” “low depression,” “moderate depression,” and “severe depression”). Continuous, quantitative scales, on the other hand, are used in 19/201 (9.5%) papers when scores are computed based on reference questionnaires (see paragraph below). Finally, 9/201 (4.5%) papers provide symptom-level predictions by focusing on individual items of aforementioned questionnaires, emotions, or other behavioral clues.

#### Reference Diagnostic Tools or Methods Used

Only 64/201 (31.8%) articles explicitly mention their reliance on standard psychiatric tools and classifications for condition assessment of the included participants. A total of 10/201 (5%) studies refer to the *DSM* (*Diagnostic and Statistical Manual of Mental Disorders*), and 11/201 (5.5%) to the *ICD* (*International Classification of Diseases*). A range of questionnaires are also mentioned, including the PHQ (30/201, 14.9%) with 8 (PHQ-8) or 9 items (PHQ-9) [75], the Hamilton Depression Scale [76] (6/201, 3%) and the Beck Depression Inventory [77] (6/201, 3%) for depression, as well as the posttraumatic stress disorder checklist for civilians [78] (3/201, 1.5%), or the Mini-Mental State Examination [79] (3/201, 1.5%) for cognitive impairment. It should be noted that the authors use such questionnaires in various ways: sometimes, scores obtained from participants’ self-administration are used as readily available labels, while in other cases the questionnaire is used to structure interviews whose transcripts will be used as input to the prediction system. In 137/201 (68.2%) studies, the authors rely on other custom methods for condition identification, or do not specify the means by which their data were annotated. These custom methods can involve asking annotators to classify texts based on the presence of certain keywords or on social media–based heuristics (eg, when texts are extracted from specific discussion forums). In 17/201 (8.5%) studies, the authors specify that labels were provided by trained clinicians.

#### Mentions of Bias and Clinical Utility–Related Themes

Mentions related to the step of outcome definition are found in 45/201 (22.4%) papers. The validity of the proxy used as a marker of a given mental health condition is questioned in 38/201 (18.9%) papers, which stress the limitations of relying on binary labels or self-report statements expressed on social media. For instance, Ohse et al [80] note that the absence of a differential diagnosis may limit the validity of their findings (derived from self-report measures), as their ground truth may have been overly inclusive. Annotation bias is also mentioned in 8/201 (4%) papers, which comprises acknowledgments that low interannotator agreement or stereotypical bias from the annotators may hinder the validity of the labels used.

### Step 4: Model Development

#### Overview

The step of model development raises the following questions: which LLMs are used? How are they evaluated?

#### LLMs Used

A total of 112 distinct LLMs are identified in our corpus, with an average count of 2.5 LLMs used per study (range: 1‐14). As detailed in Table 2, most studies rely on encoder-only models mainly derived from BERT [35] and related models such as RoBERTa [81], DistilBERT [82], and others. These models are typically used to produce contextual representations of input sequences, which are later fed to classification layers or to other models for mental health prediction. Decoder-based models derived from GPT [39] and XLNet [83] as well as encoder-decoder models are used to a lesser extent; researchers then tend to use them for prediction in a few-shot setting or, more rarely, for data augmentation or explanation generation. In 38/201 (18.9%) papers, at least one LLM which was previously trained on clinical or mental health–related content is used. We note, however, that in the remaining 163/201 (81.1%) papers, general models are preferred, which can be used off-the-shelf and optionally trained on domain data. When working on languages other than English, authors explicitly state the need to use multilingual or specialized monolingual models such as BERT derivatives for the Chinese or Arabic language.

**Table 2.** List of frequently used models in our corpus (with cutoff frequency of 4 papers)^a^.

Model	Clinical or mental health training data	Paper count, n (%)
BERT^b^ [35]	—^c^	124 (61.7)
RoBERTa [81]	—	49 (24.4)
DistilBERT [82]	—	25 (12.4)
MentalBERT [84]	Reddit mental health posts	21 (10.4)
ALBERT [85]	—	15 (7.5)
GPT-3.5	—	15 (7.5)
XLNet [83]	—	15 (7.5)
GPT-4 [86]	—	10 (5)
GPT or ChatGPT	—	10 (5)
XLM-RoBERTa [87]	—	9 (4.5)
BERT-chinese [35]	—	9 (4.5)
MentalRoBERTa [84]	Reddit mental health posts	7 (3.5)
SBERT [88]	—	6 (3)
mBERT [35]	—	6 (3)
BioClinicalBERT [89]	Clinical notes (from MIMIC-III)	5 (2.5)
MPNET [90]	—	5 (2.5)
ELECTRA [91]	—	5 (2.5)
DeBERTa [92]	—	5 (2.5)
AraBERT [93]	—	4 (2)
BioBERT [94]	Biomedical literature (PubMed, PMC)	4 (2)
DepRoBERTa [95]	Reddit mental health posts	4 (2)

a Indicates cases where the precise version of the GPT model used is not disclosed.

b BERT: Bidirectional Encoder Representations from Transformers.

c No clinical or mental health training data have been performed for these models.

#### Reported Metrics and Human Evaluation

Standard classification metrics such as accuracy, precision, specificity, recall (sensitivity), F-measure, area under the receiver operating characteristic curve, or raw confusion matrices are reported in 187/201 (93%) papers. Similarly, standard regression metrics such as mean squared error, mean absolute error, or correlation coefficients (eg, Pearson *r*) are reported in 25/201 (12.4%) papers. A range of more specific metrics used to fit the constraints of particular prediction setups was also identified: for instance, the early risk detection error [67] is designed to take into account both the correctness of a system’s binary decision and the delay needed to make that decision (measured by the number of text items seen before providing an answer). On the other hand, human-led, qualitative evaluation is very rare in the corpus. As an example, Wang et al [96] ask 50 medical interns specializing in mental illness to rate the safety, usability, and fluency of their depression detection system on a 1‐10 scale.

#### Mentions of Bias and Clinical Utility Related Themes

Mentions related to the step of model development are found in 69/201 (34.3%) papers. The main reported theme is that of the technical limitations of models (56/201, 27.8%), which can have an effect on their downstream clinical utility, although this consequence is hardly ever made explicit by the authors. These limitations include small context window size (making the processing of long documents challenging), nondomain training, an elevated computational and time cost, the need for large training datasets, etc. In addition, 13/201 (6.5%) papers explicitly mention the notion of model bias, that is, bias intrinsically encoded and amplified by LLMs. Group fairness is evoked in 5/201 (2.5%) papers, some of which report disaggregated results based on sociodemographic features (eg, age and gender [97] and sex, race, and ethnicity [48,56]).

### Step 5: Postdeployment Considerations

#### Overview

The step of postdeployment considerations raises the following questions: which measures are implemented to ensure a fair deployment? For which use cases?

#### Intended Use Cases

Many papers do not propose an explicit, concrete use case for their work. Instead, some express the general ambition to facilitate the early detection of mental health issues in individuals (57/201, 28.4%). As for more precise applications, some researchers propose to use clues in patients’ medical data to anticipate the possible onset of mental health issues (eg, depression in patients with cancer [48,56]). Others evoke the use of NLP methods to monitor global mental health trends in social media (eg, postpartum depression [98] and suicide ideation [99]), sometimes even suggesting that users at risk should be recommended to mental health specialists [59] or identified for real-world interventions [100]. Some proposals are also designed to fit more directly into the patient-caregiver relationship. Notably, Diniz et al [55] developed a web app for doctors to monitor suicidal ideations of patients estimated from their smartphone keyboard data, while Shimamoto et al [101] proposed to automatically estimate depression severity based on oral responses to an automated version of the Montgomery-Åsberg Depression Rating Scale.

#### Validation in Clinical Settings Procedures

The corpus contains no description of rigorous, systematic validation procedures in realistic clinical settings of the reviewed automated systems for mental health prediction. This could be explained by the fact that a large number of studies are retrospective, that is, they use existing data from patients the authors did not interact directly with. In that case, there is consequently no downstream impact on the concerned stakeholders, and the clinical utility is minimal. To our knowledge, none of the reviewed systems has been deployed in routine mental health care.

#### Mentions of Bias and Clinical Utility–Related Themes

Mentions related to the step of postdeployment considerations are found in 31/201 (15.4%) papers. A total of 26/201 (12.9%) papers evoke the performance of their system in the context of a future possible deployment; both positive and negative judgments are emitted. As an illustration, Matero et al [102] explicitly warn that “at this time [they] do not suggest [their] model(s) be used in practice to label mental health states,” whereas Bartal et al [50] are more confident that their model “has the potential to fit seamlessly into routine obstetric care.” Others anticipate whether the generalizability (4/201, 2%) of their system is sufficient or whether it may be confronted with integration challenges (4/201, 2.0%). Interestingly, Khalil et al [103] suggest that federated learning (ie, “an alternative that leaves the training data distributed on the mobile devices, and learns a shared model by aggregating locally-computed updates” [104]) could address issues of data privacy and regulation disparities between countries when making mental health predictions in a multilingual setting.

## Discussion

### Principal Findings

With this study, we introduced a framework to analyze methodological bias and clinical utility throughout the development pipeline of LLM-based mental health prediction systems. Our review notably sketched the prototypical profile of such a prediction system: it focuses on depressive disorders, leverages English data collected from social media, and uses BERT-based classifiers in the absence of a clinically useful outcome for the included participants. In what follows, we argue that this is problematic for multiple reasons.

### LLM-Based Mental Health Prediction Suffers From Bias All Along the Development Pipeline

First, the focus on depressive disorders leaves nearly untouched other condition families, echoing the observations of Wang et al [26]. Although it is true that they account for a large part of the world’s mental health burden, millions of individuals are also affected by anxiety disorders, bipolar disorder, schizophrenia, or eating disorders, to name a few [105]. Besides, it seems that communication disorders (affecting individuals’ language abilities) could also particularly benefit from NLP inputs [106]. In addition, despite a relative diversity among affiliation countries, papers almost exclusively work on English data, which exacerbates the existing bias toward English-based systems in NLP [29]. This bias often goes unnoticed, with nearly half of papers working on English omitting to mention it explicitly, a phenomenon that has been otherwise measured in NLP conferences [107,108]. In order to benefit a wider range of individuals, non-English or multilingual approaches should be considered.

Second, the data used in the studies largely come from social media (which was also observed by Wang et al [26]), even more so when no domain authors are involved. Some authors highlight the ease of access and collection of such data (as opposed to “traditional” clinical data), its alleged relevance to the task of mental health detection, and its wide accessibility (eg, “social media’s ubiquity presents a platform for individuals to express their feelings, instead of traditional, formal clinical settings, with 8 out of 10 people disclosing their suicidal thoughts and plans.” [109]). However, social media users do not make up representative samples of individuals, and it is difficult to ensure the quality of social media posts [30]. This is furthermore problematic as demographic information is generally absent from these datasets, and users are rarely able to provide consent for the collection of their data. In order to avoid harming users, social media research should comply with ethical guidelines as regards consent of participants, data deidentification, and sharing [110].

Third, the proxies used to account for participants’ alleged mental health status lack clinical grounding. It is unlikely that heuristics based on keywords within texts or self-administered questionnaire scores would be considered reasonable indicators by clinicians to validate diagnostic labels. This leads us to question the maturity of the field for clinical integration.

Fourth, authors tend to overlook practical requirements when adopting a model. We note that popular, nonspecialist models (and, increasingly, generative LLMs) are generally preferred, yet these choices are rarely clinically motivated. It is currently debated whether specialist models can actually be competitive with bigger, more recent generalist models [111] on specialized downstream tasks. However, the size and eventual proprietary status of the latter hinder nonnegotiable needs of data privacy and control over computational cost in clinical environments with limited resources, along with issues of environmental impact and models’ propensity to bias [112,113].

Finally, there is an overall lack of awareness of the ethical issues implied by a possible clinical deployment of the considered systems. Importantly, some works seem not to aim for such clinical use cases and deny that their systems should be used as diagnostic tools already. Yet this leads to questioning the clinical utility of tasks confined to computer science laboratories, whose results may be mistakenly interpreted as clinical conclusions. Overall, authors mainly initiate discussion themes that focus on generic ML issues related to model constraints at the model development step (eg, input length constraints, computational cost seen as a practical barrier and not an environmental problem), while revealing a data-centric view of bias around the data collection step (eg, calling for bigger, class-balanced datasets). This technical approach to bias, which calls for technical solutions, has been previously identified as the engineering ethos [114]. However, such complex issues as biases cannot be solved with a sole technique [12], if at all [18]. In what follows, we argue that an ethical approach to LLMs applied to the domain of mental health would benefit from more interdisciplinarity.

### Interdisciplinarity Is Needed to Increase Clinical Validity and Utility

In addition to the biases identified above, our observations raise the question of interdisciplinarity and its influence on the clinical utility of proposed solutions. Indeed, while we stressed global shortcomings, it should be acknowledged that studies were more robust in terms of disorder diversity, specificity of disorder definition, and quality of data sources (clinical interviews and electronic health records) when at least one author had a medical affiliation. This aligns with previous findings that constituting interdisciplinary teams (including clinicians and computer scientists) improves results validity in ML problems [115].

For instance, clinical expertise is needed to adequately define what is referred to as “depression” in studies. Some papers ambiguously assimilate the concepts of “depression” and “negative mood/sentiment” (eg, “the task is to discover the mood of the user” [116]), or “depression” and “suicidal risk” (eg, Wang et al [117] guidelines for depression level estimation are actually based on reports of suicidal ideation and plan), or do not seem to make a conceptual difference between self-diagnosed depression and clinical diagnoses produced by health care professionals. The task of disambiguating what is designated by “depression” is all the more complex because according to MeSH definitions, it can cover a sign or symptom (“Depression” [118]) or a disorder with different levels of severity (“Depressive Disorder” [119] or “Major Depressive Disorder” [120]). These differences are rarely described or implemented in data annotation protocols in studies, even though they play a crucial role in diagnostic reasoning and patient care. Consequently, training a system to automatically detect “depression” and claiming it is ready for use in clinical practice is suboptimal at best (what kind of “depression” does the system detect?), and misleading if users are left to make their own assumption of what concept of “depression” is operationalized. The close collaboration between computer scientists and medical doctors is thus required to ensure and to propagate the clinical value of the targeted outcome.

Interdisciplinary collaborations between diverse fields can also improve the robustness of systems’ evaluation. Indeed, while some studies warrant caution before real-world deployment, others present their system as deployment-ready (even in the absence of such validation), while a majority do not address the question at all. Notably, no included study presented a rigorous clinical validation for an LLM-based prediction system to be applied in practice, for example, through a randomized controlled trial [20].

Extending even beyond sole clinical validation, interdisciplinary frameworks for the validation of digital devices in medicine have recently been published. For instance, the certification of digital devices—and particularly those embarking LLMs—by the US Food and Drug Administration or the European Medicines Agency requires them to comply with the criteria of the V3 framework [121]. This framework requires rigorous clinical validation (ie, demonstrating that the metrics computed by the device correlate with meaningful physiological or clinical dimensions), but also rigorous hardware verification (validation of the sensor, if there is one) and analytical validation (ie, demonstrating that the algorithm indeed measures a behavior or a physiological marker).

This validation, however, does not ensure that the device, in its context of use, will be useful. Indeed, for most of the articles included in this review, the motivation for the research often remains vague, although untested potential use cases are described, such as large-scale mental health screening or patient monitoring. For others, very few contextual elements help grasp the medical stakes, which we believe have to be mentioned in such research. Is this to say that mental health prediction could become yet another LLM-equipped task, ensuring reasonable chances of getting a paper published?

Interestingly, an updated version of the V3 framework has been recently introduced: the V3+ framework, completing the 3 previous validations by a criterion of usability validation [122]. Although it differs from clinical utility, the addition of utility validation incorporates considerations regarding the projected use, the target population, and the intended context into the validation of digital health devices. Clarifying these elements, which requires interdisciplinary consultation between computer scientists (regarding technical validity) and clinicians (regarding usage), would thus prevent misunderstandings between the two communities, thereby avoiding empty promises fueled by each community’s overclaims regarding the capabilities of the other—particularly regarding LLMs’ ability in clinical context [123].

### Limitations

While this scoping review focuses on 201 papers published between 2019 and 2024, this period covers both the introduction of masked (eg, BERT) and autoregressive (eg, GPT) language models, which provides an opportunity to observe how they were used for mental health prediction early on. In addition, we believe that the large number of included studies allows us to provide a representative snapshot of the field in this 6-year window, through an original joint analysis of bias and clinical utility. While more recent articles could have been included, we assume that they would only have had a marginal effect on the trends we evidenced. Notably, Reiter [20] recently reported that as of March 2025, only 0.1% of papers from the ACL Anthology provided any form of downstream impact evaluation, echoing our findings in the case of mental health prediction.

Our approach is that of a scoping review, aiming at presenting a representative view of what researchers or clinicians may encounter when looking for LLM-based mental health prediction works. As such, we did not perform quality assessment of the articles at the screening stage [124]. In addition, due to the large number of included studies, we could not ensure an independent extraction by multiple reviewers, which may introduce bias in the presented results: as for other phases of the review, this was mitigated with regular discussions and methodological refinements. Since we considered a large number of qualitative entities, methodological choices were necessary to extract them into quantitative metrics. For instance, authors were labeled as “domain authors” whenever they reported being affiliated with a medical institution, a medical university department or school, or a company providing medical devices or services. While we acknowledge this to be an imperfect proxy for medical expertise, our data are freely available in Multimedia Appendices 1–4 for interested researchers to replicate our results with different methodological choices.

Finally, this study was conducted by a group of French, White, NLP researchers, including some with a background in medical informatics or psychiatry. Although we have aimed at adopting an open and interdisciplinary vision, taking into account issues related to bias and clinical utility, we may have missed some relevant aspects to these questions, starting with the phase of article identification. Because this is the main language used for research dissemination, we reviewed only articles written in English in major scientific databases, which nonetheless allowed us to retrieve studies working on other languages. We intentionally crafted broad queries to minimize false negatives, and considered multiple literature sources relevant to NLP and medical sciences. Yet, different requests and additional sources may retrieve slightly different results. Also, information about eventual clinical deployments of the considered systems outside of what is reported in the papers may have been missed.

### Broader Implications

This review focused solely on LLM-based mental health prediction. We did not consider interventional applications (eg, chatbots for mental health support), nor other medical domains, and some of our observations may not generalize. Nevertheless, we believe that some of our findings are part of a broader picture. Our study suggests that over the past 6 years, the perspective of downstream usage (and users) has seldom been considered in the development of LLM-based, predictive mental health applications. This echoes trends that have been reported in NLP [20]. The overemphasis on algorithm development [125] and quantitative, benchmark-based competition for performance [126] poses a risk of harm by assuming medical applications are “yet another task” to tackle despite increased ethical risks. Finally, it would be interesting to study more deeply the “promises” and narratives around AI and NLP for (mental) health care (eg, the promise to assist health care workers without replacing them, the promise to be cost-effective, and so on), which is left for future research.

### Conclusions

LLMs are increasingly used in mental health prediction tasks. Following a 5-step pipeline as a framework to analyze NLP proposals, we described an emerging field that exhibits some marked tendencies, notably toward the detection of depressive disorders and the use of social media data in English. We showed that researchers are generally aware of some bias and clinical utility–related issues, but that reports in papers are sparse and not systematic. In particular, postdeployment considerations and impact studies are lacking, which leads us to question the idea that LLMs have the potential to revolutionize mental health care. When dealing with NLP-assisted mental health research, we advocate for a combined view of bias and clinical utility, implying that no biased system can be clinically useful, and vice versa. This requires interdisciplinarity and careful efforts all along the research pipeline. We strongly believe that the goal to contribute to mental health research and, ultimately, to benefit patients should remain central in NLP efforts towards mental health.

## Supplementary material

10.2196/88082 Multimedia Appendix 1 Search strategy for studies inclusion with full string requests.

10.2196/88082 Multimedia Appendix 2 Extraction guide detailing the entities extracted, the rationale for inclusion, and operationalization.

10.2196/88082 Multimedia Appendix 3 Table of extracted and analyzed entities from the reviewed papers.

10.2196/88082 Multimedia Appendix 4 Complete list of included studies with metadata.

10.2196/88082 Checklist 1 PRISMA-ScR checklist.

## Abbreviations

BERT: Bidirectional Encoder Representations from Transformers
*DSM*: *Diagnostic and Statistical Manual of Mental Disorders*
*DSM-5*: *Diagnostic and Statistical Manual of Mental Disorders, Fifth Edition*
*ICD*: *International Classification of Diseases*
LLM: large language model
ML: machine learning
NLP: natural language processing
PHQ: Patient Health Questionnaire
PHQ-8: Patient Health Questionnaire-8
PHQ-9: Patient Health Questionnaire-9
PRISMA: Preferred Reporting Items for Systematic Reviews and Meta-Analyses
PRISMA-ScR: Preferred Reporting Items for Systematic Reviews and Meta-Analyses extension for Scoping Reviews
