# Supplementary material for: Large Language Models for Mental Health Prediction: Scoping Review of Bias and Clinical Utility Documentation in 2019-2024
Source: JMIR AI. 2026 Aug 13;5:e88082. doi: 10.2196/88082 (PMC13472528; doi:10.2196/88082)
Supplement: Supplementary file 1 [file ai-v5-e88082-s002.pdf]

Extraction Guide

The extraction was performed on full-text articles. First, entities related to the methodology adopted by authors in their work were extracted. Second, authors' self-statements related to bias and clinical utility were extracted.

This documents lists all extracted entities, providing a description, a rationale for extraction (referring to relevant sections of the works of [Hovy and Prabhumoye, 2021] and [Chen et al., 2021] which inspired our methodology) and their operationalization by specifying accepted values. Entities with a row entitled 'accepted_values_agg' (instead of 'accepted_values') are those for which we aggregated initial sparse results into meaningful categories (thematic coding).

NB : In what follows, references [Hovy and Prabhumoye, 2021] and [Chen et al., 2021] are referred to as [H] and [C]. '§' is used as an abbreviation for 'section'.

Authors' methodology

Step 1 - Research design and problem selection

affiliation_countries

description	All affiliation countries declared in the header of the paper
rationale	To document the diversity of research teams (§2.5 [H], §2.4 [C]) w.r.t represented countries
accepted_values	Comma-separated full names of the affiliation countries

authors_count

description	Number of authors in the reviewed paper
rationale	To be used along the number of 'domain' authors of the paper (see **domain_authors_count** below) to compute the proportion of 'domain' authors within the paper
accepted_values	<Integer>

domain_authors_count

description	Number of 'domain' authors in the reviewed paper, i.e., any author declaring a medical degree or an affiliation loosely related to the medical domain : a medical school, a medical department of a university, a hospital, a company providing medical tools/services, etc.
rationale	To document the diversity of research teams (§2.5 [H], §2.4 [C]) w.r.t interdisciplinary expertise
accepted_values	<Integer>

data_language

description	All languages the authors work on for mental health prediction (i.e. What is the language of the data?)

rationale	To document the diversity of served languages (§2.5 [H])
accepted_values	Comma-separated ISO 639-1 codes of the corresponding langages (e.g. 'en', 'zh', etc.)

explicit_data_language

description	Whether the authors explicitly state the language(s) they are working on
rationale	To measure implicit assumptions that some languages (typically English) are 'default' languages in research (§2.5 [H])
accepted_values	<Boolean><br>• **True** : the languages are explicited in the text of the article<br>• **False** : the languages have to be deduced from the models or resources that the authors use

studied_conditions

description	All mental health conditions that the authors declare they predict with LLMs
rationale	To measure the distribution of research efforts in the field of mental health (§2. [C])
accepted_values_agg	Comma-separated conditions from the following list of conditions / groups of conditions described in the DSM-V:<br>• '**depression**'<br>• '**suicide**' # for further study in DSM-V<br>• '**trauma**' (includes PTSD)<br>• '**schizophrenia**'<br>• '**anxiety**'<br>• '**alzheimer**'<br>• '**adhd**'<br>• '**bipolar**'<br>• '**eatingd**' (eating disorders)<br>• '**intellectual-disabilities**'<br>• '**autism**'<br>• '**dissociation**' (dissociative disorders)<br>• '**personality**' (personality disorders)<br>• '**ocd**' (obsessive-compulsive disorders)

pb_selection_rationale

description	Elements of context provided by the authors as rationale for studying the considered mental health condition(s) / for making LLM-based mental health prediction.
rationale	To document the motivations underlying problem selection choices and to estimate their connection with clinical needs (§2. [C])
accepted_values_agg	Comma-separated list of values from the following list :<br>• '**shared-task**' : the authors make it explicit that their work is part of a shared task or challenge in a scientific event<br>• '**impact'** : the authors highlight the negative impacts of the considered mental health condition(s) on people<br>• '**prevalence'** : the authors highlight that the prevalence of the considered mental health condition(s) is high or rising<br>• '**tradi-limitations**' : the authors highlight limitations of the

	traditionnal health system with regards to the diagnosis/treatment of the considered mental health condition(s) ; e.g. they evoke the subjectivity of doctors, the limitation of existing diagnosis tools, etc.

Step 2 - Data collection

**data_sources**	
description	Data sources used by the authors for training and/or evaluation of their mental health prediction system
rationale	To document the representativeness and clinical relevance of the included data sources (§2.1[H], §3 [C])
accepted_values_agg	Comma-separated codes for each of the following categories, possibly indicating a finer-level of details with square brackets (e.g. 'sm[reddit]') :<br>• '**sm**' : texts posted by users on social media or online forums<br>○ 'reddit', 'twitter', 'sina-weibo', 'other'<br>• '**itw**' : data purposely collected during an interview (possibly a therapy session) with the included participants<br>• '**ehr**' : electronical health records<br>• '**other**' : data sources not fitting the categories above

**report_num_participants**	
description	Whether the authors report the number of people represented in at least one dataset that they use
rationale	To document minimal effort in describing the population whose data was used to perform the study (§2.1[H], §3.2 [C])
accepted_values	<Boolean>

**report_num_docs**	
description	Whether the authors report the number of documents contained in at least one dataset that they use
rationale	To document minimal effort in describing the data which was used to perform the study (to be compared with **report_num_participants** above) (§2.1[H], §3.2 [C])
accepted_values	<Boolean>

**reported_demo_chars**	
description	List of reported socio-demographic attributes in at least one dataset used by the authors
rationale	To document intermediate effort in describing the population whose data was used to perform the study (§2.1[H], §3.2 [C])
accepted_values_agg	Comma-separated values among the following list :<br>• '**age**'<br>• '**sex_gender**'<br>• '**race_ethnicity_cultural_background**'<br>• '**education**' (level of education or academic status)<br>• '**other**' (including marital status, income level, insurance status,

or medical information)

report_technical_details

description — Whether the authors report any ‘technical’ detail about at least one dataset that they use. Technical details comprise : the class-distribution for categorical data (e.g. the number of positive vs. negative cases), statistics for continuous data (e.g. the average PHQ-8 score in the dataset) , and the year of collection of the data.

rationale — To document intermediate effort in describing the data which was used to perform the study (to be compared with **reported_demo_chars** above) (§2.1[H], §3.2 [C])

accepted_values — <Boolean>

Step 3 - Outcome definition

outcome_modalities

description — Outcome modalities of the mental health prediction task(s) as modelled in the reviewed paper.

rationale — To describe the typical output of the developed predictive system (to be discussed in relation with what constitutes clinically actionable results) (§4 [C])

accepted_values_agg — Comma-separated list of values :

- ‘**binary**’ (e.g. *depression* or *no depression*)
- ‘**severity-scale**’ (ordinal, qualitative scale, e.g., *low* or *moderate* or *severe depression*)
- ‘**severity-score**’ (quantitative scale typically linked with a questionnaire, e.g. score between 0-24)
- ‘**symptoms**’ (the authors only predict individual symptoms instead of the condition(s) itself)

gold_references

description — Reference taxonomies or diagnostic tools referred to by the authors, either because they were used to constitute their gold data, or because they rely on them to process the data and perform the prediction.

rationale — To analyze the recognized validity and limitations of the operationalized proxies (§4 [C], §2.2 [H])

accepted_values — Comma-separated list of the tools (e.g., ‘PHQ-8’, ‘DSM-5’, etc.)

Step 4 - LLM development and evaluation

models

description — List of all LLM models used in the reviewed paper

rationale — To inventory all LLMs used (to be discussed in relation with model bias and clinical relevance of the chosen models) (§2.3-2.4 [H])

accepted_values — Comma-separated list of the LLM names (e.g. ‘bert’, ‘gpt-3.5’, etc.)

automated_metrics

description	All the metrics the authors use to report the performance of their system (automated evaluation)
rationale	To investigate the limitations and clinical meaningfulness of the chosen performance metrics (§5.4 [C])
accepted_values	Comma-separated list of the metrics (e.g., ‘precision’, ‘F1-score’)

disaggregated_evaluation

description	Whether humans the authors provide disaggregated evaluation results where the performance is assessed not only on the entire set of participants but also on sub-groups (e.g., men vs. women).
rationale	To document whether group fairness is computed (§5.4-5.5 [C])
accepted_values	<Boolean>

human_evaluation

description	Whether humans evaluate the output of the system.
rationale	To account for the part of human control in the quality of the system.
accepted_values	<Boolean>

Step 5 - Post-deployment considerations

use_cases

description	Envisioned or readily-deployed use cases for the systems developed by the authors. For instance : social media monitoring, early detection of mental health conditions, creation of a mental health tracking app, psychiatrist assistance, etc.
rationale	To document the use cases reported by the authors and later discuss their feasibility and adequation with clinical needs.
accepted_values	Free text (this entity is difficult to cut into predefined labels, so feel free to mention use cases that do not belong in the provided list of examples).

clinical_validation

description	Whether a clinical validation of the system has been done
rationale	To assess the conformity with clinical expectations (§6.1 [C], §2.5 [H])
accepted_values	<Boolean>

Authors’ self statements about bias / clinical utility

In this part, you have to fill 5 columns, each one corresponding with one step of the NLP system pipeline (e.g., **bias_pb_selection**).

At a given pipeline step, you’ll have to track a certain number of themes (detailed below). If a theme is mentioned in the paper, keep track of one sentence/paragraph which illustrate it, and write

it in the column as a tuple in the following format : (‘theme’, ‘sentence’). If multiple themes are found, the column should contain a comma-separated list of tuples.

Step 1 - Research design and problem selection

The themes proposed for this step relate to the reflexions emitted in §2 [C] and §2.5 [H].

theme	description
**research_gap**	The authors acknowledge that the condition they are working on < is bridging / is perpetuating > a research gap (i.e., the fact that some mental health conditions are disproportionately more studied than others).
**population_disparities**	The authors explicitly state that they < focus on / omit > a part of the population which is more < vulnerable to / affected by > the studied condition(s).
**language_culture**	The authors explicitly state that they < focus on / omit > a part of the population by focusing on a given langage or cultural setting.
**workforce_diversity**	The authors explicitly state that their team < is diverse / lacks diversity > regarding sociodemographic attributes (gender, race/ethnicity, etc.) or professional background (clinicians, computer scientists, etc.)

Step 2 – Data collection

The themes proposed for this step relate to the reflexions emitted in §3 [C] and §2.1 [H].

theme	description
**class_imbalance**	The authors acknowledge that the data they work with < is imbalanced / was corrected for imbalance >, with regards to the studied condition (e.g. if there is a majority of negative cases) OR they call for more balanced data in future studies.
**representation_bias**	The authors acknowledge that the data they work with < is not representative / was corrected for representativeness > of the target population, with regards to the demographic attributes of the included participants OR they call for more representative data in future studies.
**small_dataset**	The authors acknowledge that a dataset they work with is small with regards to the expectations of their experimental setup.
**pre_existing_bias**	The authors acknowledge that the data they work with <may contain / has been checked> for pre-existing bias (i.e., already present at the time of collection).
**static_dataset**	The authors state that the data they work with <is / is not> limited because it contains data from a single point in time instead of longitudinal data (e.g. patient records spanning multiple hospital visits).

Step 3 – Outcome definition

The themes proposed for this step relate to the reflexions emitted in §4 [C] and §2.2 [H].

theme	description
**label_validity**	The authors acknowledge that their findings may be limited by the labels

	they chose as a reference for the mental health status of participants (e.g. binary labels, self-report instead of clinically validated diagnoses, etc.)
**annotation_bias**	The authors acknowledge that the data they work with <may contain / has been checked> for annotation bias (i.e., bias introduced by the annotators)

Step 4 – LLM development

The themes proposed for this step relate to the reflexions emitted in §4 [C] and §2.4 [H].

**theme**	**description**
**model_limitations**	The authors acknowledge that their system < suffers / may suffer > from ‘technical’ limitations of the LLMs used : hallucinations, computational costs, proprietary nature, non-explainability, non-reproducibility, etc.
**model_bias**	The authors acknowledge that their system < suffers / may suffer > from intrinsic LLM bias (i.e., bias encoded, and potentially amplified by LLMs)
**performance_fairness**	The authors explicitly take into account disaggregated evaluation results or stress possible performance disparities among population subgroups.
**metrics**	The authors stress that the metrics they chose < may be limited / were purposely chosen > to reflect clinically meaningful results.

Step 5 – Postdeployment considerations

The themes proposed for this step relate to the reflexions emitted in §5 [C] and §2.5 [H].

**theme**	**description**
**performance**	The authors make explicit statements about the extent to which their system’s performance is sufficient for deployment in ‘real-life’ scenarios.
**generalization**	The authors make explicit statements about the extent to which their system would be able to generalize in ‘real-life’ scenarios.
**consent_privacy**	The authors explicitly state that issues related with patient consent or privacy < could emerge / have been anticipated > in possible deployments of their system.
**integration_challenges**	The authors explicitly state that issues related to the ‘real-life’ integration of their system < could emerge / have been anticipated >.

References

Hovy D, Prabhumoye S. Five sources of bias in natural language processing. Language and Linguistics Compass. 2021;15(8):e12432. doi:10.1111/lnc3.12432.

Chen IY, Pierson E, Rose S, Joshi S, Ferryman K, Ghassemi M. Ethical Machine Learning in Healthcare. Annual Review of Biomedical Data Science. 2021 Jul 20;4(Volume 4, 2021):123–44. doi:10.1146/annurev-biodatasci-092820-114757
