# Supplementary material for: Large Language Models for Mental Health Prediction: Scoping Review of Bias and Clinical Utility Documentation in 2019-2024
Source: JMIR AI. 2026 Aug 13;5:e88082. doi: 10.2196/88082 (PMC13472528; doi:10.2196/88082)
Supplement: Supplementary file 3 [file ai-v5-e88082-s001.docx]

Request details

All searches were performed on January 20, 2025.

# PubMed

- <https://pubmed.ncbi.nlm.nih.gov/advanced/>

- 1525 records identified

(("suicid*"[tiab] OR "psycho*"[tiab] OR "mental health"[mh] OR "mental health"[tiab] OR "depress*"[tiab] OR "anxiety"[tiab] OR "psychiatr*"[tw] OR "mental illness*"[tiab] OR "telemental health"[tiab])

AND

("natural language processing"[tiab] OR "language model*"[tiab] OR "transformer*"[tiab] OR "pretrained"[tiab] OR "llm"[tiab] OR "llms"[tiab] OR "chatbot*"[tiab] OR "conversational agent*"[tiab] OR "bert"[tiab] OR "gpt"[tiab] OR "chatgpt"[tiab] OR "bard"[tiab] OR "palm"[tiab] OR "llama"[tiab] OR "alpaca"[tiab] OR "vicuna"[tiab])

AND

("predict*"[tiab] OR "identif*"[tiab] OR "classif*"[tiab] OR "categori*"[tiab] OR "detect*"[tiab] OR "triag*"[tiab] OR "diagnos*"[tiab] OR "assess*"[tiab] OR "extract*"[tiab] OR "retriev*"[tiab] OR "decision making"[tiab]))

NOT

(("preprint"[pt] OR "editorial"[pt] OR "letter"[pt] OR "review"[pt] OR "meta-analysis"[pt]) OR ("review"[title]))

AND

(2019/1/1:2024/12/31[pdat] AND ("English"[language]))

# ACM Digital Library

- <https://dl.acm.org/search/advanced>

- 36 records identified

((Abstract: ("suicid*") OR Abstract: ("psycho*") OR Abstract: ("mental health") OR Abstract: ("depress*") OR Abstract: ("anxiety") OR Abstract: ("psychiatr*") OR Abstract: ("mental illness*") OR Abstract: ("telemental health") OR Title: ("suicid*") OR Title: ("psycho*") OR Title: ("mental health") OR Title: ("depress*") OR Title: ("anxiety") OR Title: ("psychiatr*") OR Title: ("mental illness*") OR Title: ("telemental health")) AND (Abstract: ("natural language processing") OR Abstract: ("language model*") OR Abstract: ("transformer*") OR Abstract: ("pretrained") OR Abstract: ("llm") OR Abstract: ("llms") OR Abstract: ("chatbot*") OR Abstract: ("conversational agent*") OR Abstract: ("bert") OR Abstract: ("gpt") OR Abstract: ("chatgpt") OR Abstract: ("bard") OR Abstract: ("palm") OR Abstract: ("llama") OR Abstract: ("alpaca") OR Abstract: ("vicuna") OR Title: ("natural language processing") OR Title: ("language model*") OR Title: ("transformer*") OR Title: ("pretrained") OR Title: ("llm") OR Title: ("llms") OR Title: ("chatbot*") OR Title: ("conversational agent*") OR Title: ("bert") OR Title: ("gpt") OR Title: ("chatgpt") OR Title: ("bard") OR Title: ("palm") OR Title: ("llama") OR Title: ("alpaca") OR Title: ("vicuna")) AND (Abstract: ("predict*") OR Abstract: ("identif*") OR Abstract: ("classif*") OR Abstract: ("categori*") OR Abstract: ("detect*") OR Abstract: ("triag*") OR Abstract: ("diagnos*") OR Abstract: ("assess*") OR Abstract: ("extract*") OR Abstract: ("retriev*") OR Abstract: ("decision making") OR Title: ("predict*") OR Title: ("identif*") OR Title: ("classif*") OR Title: ("categori*") OR Title: ("detect*") OR Title: ("triag*") OR Title: ("diagnos*") OR Title: ("assess*") OR Title: ("extract*") OR Title: ("retriev*") OR Title: ("decision making")))

# IEEE Xplore

- <https://ieeexplore.ieee.org/search/advanced>

- 818 records identified

((("Abstract":"suicide") OR ("Abstract":"suicidal") OR ("Abstract":"psych*") OR ("Abstract":"mental health") OR ("Abstract":"depression") OR ("Abstract":"depressive") OR ("Abstract":"anxiety") OR ("Abstract":"mental illness") OR ("Abstract":"mental illnesses") OR ("Abstract":"telemental health") OR ("Publication Title":"suicide") OR ("Publication Title":"suicidal") OR ("Publication Title":"psych*") OR ("Publication Title":"mental health") OR ("Publication Title":"depression") OR ("Publication Title":"depressive") OR ("Publication Title":"anxiety") OR ("Publication Title":"mental illness") OR ("Publication Title":"mental illnesses") OR ("Publication Title":"telemental health")) AND (("Abstract":"natural language processing") OR ("Abstract":"language model") OR ("Abstract":"language models") OR ("Abstract":"transformer") OR ("Abstract":"transformers") OR ("Abstract":"pretrained") OR ("Abstract":"llm") OR ("Abstract":"llms") OR ("Abstract":"chatbot") OR ("Abstract":"chatbots") OR ("Abstract":"conversational agent") OR ("Abstract":"conversational agents") OR ("Abstract":"bert") OR ("Abstract":"gpt") OR ("Abstract":"chatgpt") OR ("Abstract":"bard") OR ("Abstract":"palm") OR ("Abstract":"llama") OR ("Abstract":"alpaca") OR ("Abstract":"vicuna") OR ("Publication Title":"natural language processing") OR ("Publication Title":"language model") OR ("Publication Title":"language models") OR ("Publication Title":"transformer") OR ("Publication Title":"transformers") OR ("Publication Title":"pretrained") OR ("Publication Title":"llm") OR ("Publication Title":"llms") OR ("Publication Title":"chatbot") OR ("Publication Title":"chatbots") OR ("Publication Title":"conversational agent") OR ("Publication Title":"conversational agents") OR ("Publication Title":"bert") OR ("Publication Title":"gpt") OR ("Publication Title":"chatgpt") OR ("Publication Title":"bard") OR ("Publication Title":"palm") OR ("Publication Title":"llama") OR ("Publication Title":"alpaca") OR ("Publication Title":"vicuna")) AND (("Abstract":"predict*") OR ("Abstract":"identify") OR ("Abstract":"identification") OR ("Abstract":"classify") OR ("Abstract":"classification") OR ("Abstract":"categorize") OR ("Abstract":"categorization") OR ("Abstract":"categorise") OR ("Abstract":"categorisation") OR ("Abstract":"detect") OR ("Abstract":"detect*") OR ("Abstract":"triage") OR ("Abstract":"triaging") OR ("Abstract":"diagnose") OR ("Abstract":"diagnosis") OR ("Abstract":"assess") OR ("Abstract":"assessment") OR ("Abstract":"extract") OR ("Abstract":"extraction") OR ("Abstract":"retrieve") OR ("Abstract":"retrieval") OR ("Abstract":"decision making") OR ("Publication Title":"predict*") OR ("Publication Title":"identify") OR ("Publication Title":"identification") OR ("Publication Title":"classify") OR ("Publication Title":"classification") OR ("Publication Title":"categorize") OR ("Publication Title":"categorization") OR ("Publication Title":"categorise") OR ("Publication Title":"categorisation") OR ("Publication Title":"detect") OR ("Publication Title":"detect*") OR ("Publication Title":"triage") OR ("Publication Title":"triaging") OR ("Publication Title":"diagnose") OR ("Publication Title":"diagnosis") OR ("Publication Title":"assess") OR ("Publication Title":"assessment") OR ("Publication Title":"extract") OR ("Publication Title":"extraction") OR ("Publication Title":"retrieve") OR ("Publication Title":"retrieval") OR ("Publication Title":"decision making")))

# Web of Science

- <https://clarivate.com/products/web-of-science/> (using institutional access)

- 236 records identified

(((TI=suicid* OR AB=suicid*) OR (TI=psycho* OR AB=psycho*) OR ALL="mental health" OR (TI="mental health" OR AB="mental health") OR (TI=depress* OR AB=depress*) OR (TI=anxiety OR AB=anxiety) OR ALL=psychiatr* OR (TI="mental illness*" OR AB="mental illness*") OR (TI="telemental health" OR AB="telemental health"))

AND

((TI="natural language processing" OR AB="natural language processing") OR (TI="language model*" OR AB="language model*") OR (TI=transformer* OR AB=transformer*) OR (TI=pretrained OR AB=pretrained) OR (TI=llm OR AB=llm) OR (TI=llms OR AB=llms) OR (TI=chatbot* OR AB=chatbot*) OR (TI="conversational agent*" OR AB="conversational agent*") OR (TI=bert OR AB=bert) OR (TI=gpt OR AB=gpt) OR (TI=chatgpt OR AB=chatgpt) OR (TI=bard OR AB=bard) OR (TI=palm OR AB=palm) OR (TI=llama OR AB=llama) OR (TI=alpaca OR AB=alpaca) OR (TI=vicuna OR AB=vicuna))

AND

((TI=predict* OR AB=predict*) OR (TI=identif* OR AB=identif*) OR (TI=classif* OR AB=classif*) OR (TI=categori* OR AB=categori*) OR (TI=detect* OR AB=detect*) OR (TI=triag* OR AB=triag*) OR (TI=diagnos* OR AB=diagnos*) OR (TI=assess* OR AB=assess*) OR (TI=extract* OR AB=extract*) OR (TI=retriev* OR AB=retriev*) OR (TI="decision making" OR AB="decision making")))

NOT

((ALL=preprint OR ALL=editorial OR ALL=letter OR ALL=review OR ALL=meta-analysis) OR (TI=review))

AND

(ALL=2019 AND (ALL=English ))

# ACL Anthology

As the ACL Anthology website does not allow for advanced searches, we used the Python library acl_anthology. We provide the script used to perform the search.

- 31 records identified

```
#! pip install -q acl_anthology tqdm re
from acl_anthology import Anthology
import tqdm
import re

## define keyword groups for request
# keyword group : mental health (using * as wildcard)
kw_mh_wc = ["suicid*", "psycho*", "mental health", "depress*", "anxiety", "psychiatr*", "mental illness*", "telemental health"]

# keyword group : (large) language models (using * as wildcard)
kw_lm_wc = ["natural language processing", "language model*", "transformer*", "pretrained", "llm", "llms",
"chatbot*", "conversational agent*", "bert", "gpt", "chatgpt", "bard", "palm", "llama", "alpaca", "vicuna"]

# keyword group : detection task (using * as wildcard)
kw_dt_wc = ["predict*", "identif*", "classif*", "categori*", "detect*", "triag*", "diagnos*", "assess*", "extract*", "retriev*", "decision making"]

# load the ACL anthology content
anthology = Anthology.from_repo()
all_papers = list(anthology.papers())

# keep only papers between 2019 and 2024
to_keep = []
for paper in tqdm.tqdm(all_papers):
    if "2019" in paper.bibkey or "2020" in paper.bibkey or "2021" in paper.bibkey or "2022" in paper.bibkey or "2023" in paper.bibkey or "2024" in paper.bibkey:
        to_keep.append(paper)

# select papers containing 1+ keyword per group in their abstract or title
matches = []

for paper in tqdm.tqdm(to_keep):
    title = str(paper.title)
    abstract = str(paper.abstract)
    found = [False, False, False]

    for i, group in enumerate([kw_mh_wc, kw_lm_wc, kw_dt_wc]):
        for keyword in group:
            pattern = re.compile(f"\\b{keyword}\\b", re.IGNORECASE)
            if re.search(pattern, title) or re.search(pattern, abstract):
                found[i] = True
                break # once found, can exit this loop
            if all(found):
                matches.append(paper)

## now save the matches into a separate file
def add_abstract_to_bibtex(paper):
    bibtex = paper.to_bibtex()
    abstract = paper.abstract
    if "abstract = {" not in bibtex:
        bibtex = bibtex[:len(bibtex)-2] # remove last }
        bibtex += f',\n abstract = "{abstract}"'
        bibtex += "\n}"
    return bibtex

with open("final/acl-antho.txt", "w") as f:
    for match in matches:
        bibtex_entry = add_abstract_to_bibtex(match)
        f.write(bibtex_entry)
        f.write("\n\n")
```
